# Immunomodulation Through Low-Dose Radiation for Severe COVID-19: Lessons From the Past and New Developments

**DOI:** 10.1177/1559325820956800

**Published:** 2020-09-22

**Authors:** Yannic N. Hanekamp, James Giordano, Jaap C. Hanekamp, Mohammad K. Khan, Maarten Limper, Constantijn S. Venema, Samuel D. Vergunst, Joost J. C. Verhoeff, Edward J. Calabrese

**Affiliations:** 1 University Medical Centre Groningen, University of Groningen, the Netherlands; 2 Departments of Neurology and Biochemistry, and Pellegrino Center for Clinical Bioethics, Georgetown University Medical Center, Washington, DC, USA; 3 University College Roosevelt, Middelburg, the Netherlands; 4 Department of Environmental Health Sciences, University of Massachusetts, Amherst, MA, USA; 5 Department of Radiation Oncology, Winship Cancer Institute, Emory University School of Medicine, Atlanta, GA, USA; 6 Department of Rheumatology and Clinical Immunology, University Medical Center Utrecht, Utrecht University, Utrecht, the Netherlands; 7 Department of Radiation Oncology, University Medical Center Utrecht, Utrecht University, Utrecht, the Netherlands

**Keywords:** COVID-19, low-dose radiation, inflammation, treatment, cytokine storm

## Abstract

Low-dose radiation therapy (LD-RT) has historically been a successful treatment for pneumonia and is clinically established as an immunomodulating therapy for inflammatory diseases. The ongoing COVID-19 pandemic has elicited renewed scientific interest in LD-RT and multiple small clinical trials have recently corroborated the historical LD-RT findings and demonstrated preliminary efficacy and immunomodulation for the treatment of severe COVID-19 pneumonia. The present review explicates archival medical research data of LD-RT and attempts to translate this into modernized evidence, relevant for the COVID-19 crisis. Additionally, we explore the putative mechanisms of LD-RT immunomodulation, revealing specific downregulation of proinflammatory cytokines that are integral to the development of the COVID-19 cytokine storm induced hyperinflammatory state. Radiation exposure in LD-RT is minimal compared to radiotherapy dosing standards in oncology care and direct toxicity and long-term risk for secondary disease are expected to be low. The recent clinical trials investigating LD-RT for COVID-19 confirm initial treatment safety. Based on our findings we conclude that LD-RT could be an important treatment option for COVID-19 patients that are likely to progress to severity. We advocate the further use of LD-RT in carefully monitored experimental environments to validate its effectiveness, risks and mechanisms of LD-RT.

## Background

As the coronavirus disease COVID-19 has spread the globe, physicians are confronted with patients that progressively develop severe pneumonia as a consequence of an excessive inflammatory response against SARS-CoV-2. This hyperinflammatory state—irrespective of whether constituent to pneumonia—can contribute to acute respiratory distress syndrome (ARDS),^1^ for which only supportive treatment of supplemental oxygen and mechanical ventilation is available.^2^ These measures are often ineffective in preventing mortality (i.e.- the estimated mortality rate being approximately 41.9-50.4%).^3^ The hospitalized COVID-19 patient group comprises a minority of those afflicted with COVID-19, yet these patients require care that incurs massive impact on hospital resources, services, and personnel.

Exploring immunomodulatory treatments to mitigate the progression to ARDS in severe COVID-19 may prove lifesaving, and this, in our view, supports—if not prompts—the urgent need to develop more effective interventions for the most critically ill COVID-19 patients. We posit that in order to move forward on this exigent issue, it will be important to look back: historically, low dose of radiation has been used as immunomodulating therapy to treat pneumonia, and other inflammatory and infectious diseases.^4-10^ While the use of low dose radiation has decreased in light of the advent of antibiotics, radiation therapy continues to be employed to ameliorate other conditions with known inflammatory states (e.g.- arthritis).^11^ Herein, we present extant and newly analyzed evidence of the effectiveness of low dose radiation therapy (henceforth LD-RT), and propose a treatment schedule that we hope to be of benefit to severely ill COVID-19 patients in dire straits.

## Historical Use of LD-RT for Pneumonia

Contemporary radiation therapy is almost exclusively used in oncology and is based upon the ability of high doses of ionizing radiation to destroy mitotically active cells. Lesser known, yet clinically established, are the anti-inflammatory effects of LD-RT at doses less than 1.0 Gy (i.e.- 50 to 100 times lower than those used in oncologic care).^12,13^ Before the widespread clinical availability and use of antibiotics (i.e.- in the early 1940s) pneumonia posed a serious challenge for patients and clinicians alike. With limited therapeutics (e.g.- antibacterials like sulfonamides, which caused serious toxic side-effects) clinicians sought other options to treat pneumonia, including LD-RT. A number of studies about the use of LD-RT for pneumonia have since been published [for review, see 4]. Of particular interest, are those reports that addressed atypical/presumed-viral pneumonia and/or included control groups.

Rousseau presented an investigation of 29 atypical pneumonia patients that were unresponsive to a (3-7 days) regimen of sulfanilamide treatment.^14^ It was observed that “The patients were growing rapidly and progressively worse with adequate doses of the drug [sulfanilamide]. By all clinical standards, it appeared that death was inevitable in all cases in this group.” Fifteen (15) to 20 hours following LD-RT, 22 of the 29 patients demonstrated a full clinical recovery, as evidenced by a decreased temperature, pulse, respiratory rate and white blood cell count. The 7 remaining patients failed to respond to the treatment and died. In total, Rousseau reported on 176 pneumonia patients treated with LD-RT and provided a control mortality rate by using data on pneumonia cases of the same hospital.^14^ Of the group receiving treatment, 5.7% died, compared to the overall hospital pneumonia mortality rate of 28%. Of note is, Rousseau’s remark that “X-Ray therapy has been found strikingly free from any toxic side-effects.”

**Figure 1.**
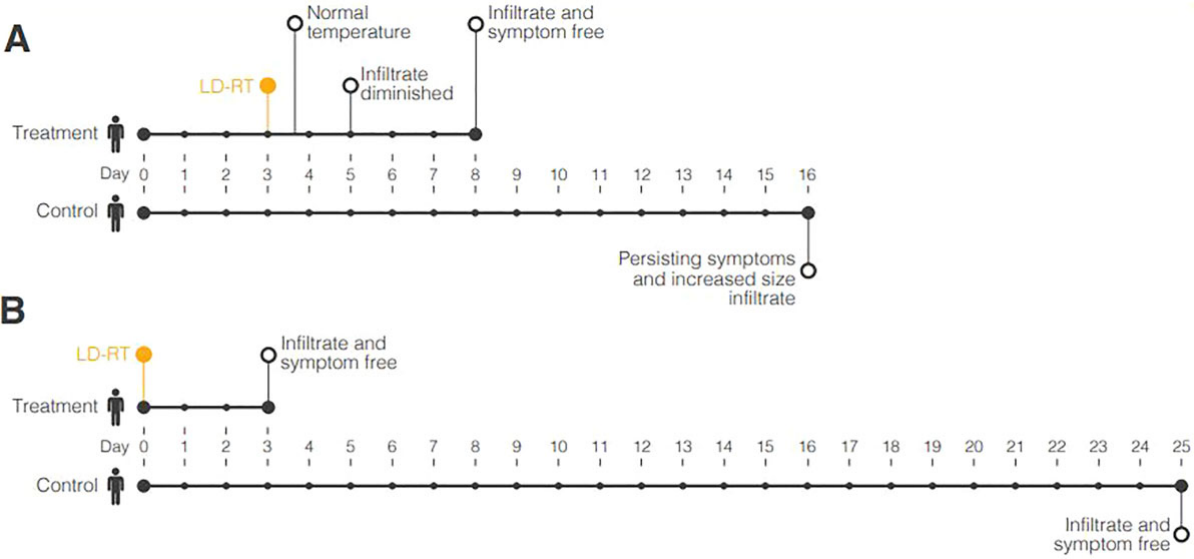
Control versus LD-RT disease-course timelines of 2 pairs of patients with presumed viral pneumonia reported. Day 0 represents the onset of disease. Timeline A illustrates the disease course of 2 paired middle-aged men (ages 56 and 64) with severe pneumonia. Timeline B illustrates the disease course of 2 paired females aged 6 with pneumonia.

Oppenheimer reported 56 presumed-viral pneumonia cases treated with LD-RT.^15^ In 45 patients, fever completely resolved, and pulmonary chest X-ray consolidations disappeared within 3-5 days after LD-RT. Control patients (of like sex, somatotype, and of similar age) did not exhibit any clinically relevant change in disease presentation or severity. These paired comparisons are illustrated in Figure 1. Of the 56 patients, 3 were reported to experience acute toxicity effects (i.e.- chills, convulsions, cold sweats) post-treatment.

In a case-series report of 231 patients with LD-RT, Powell described notable clinical success, but was not permitted to include a control group after the hospital staff recognized that treatment produced near immediate relief of respiratory and circulatory distress.^16-18^ However, Powell reported outcomes of 76 prior consecutive pneumonia cases without LD-RT; noting that only 6.9% of patients in the treatment group died, compared to 28.9% of patients in the control group. Treatment complications were only reported for the first 105 patients in the series; of these patients, 7 developed empyema, with 2 requiring surgical drainage. One patient, known to have tuberculosis and bronchiectasis, was diagnosed with a pulmonary abscess.

**Figure 2.**
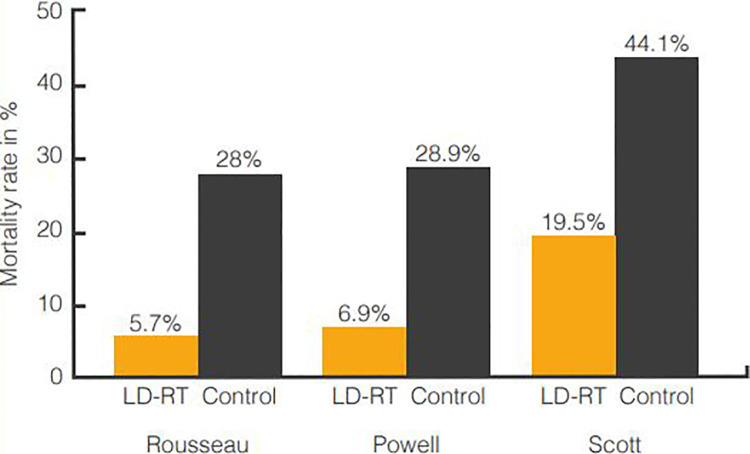
Mortality rates of pneumonia cases in LD-RT and control groups reported in studies by Rousseau, Powell and Scott.^16,18-21^

In a report describing 138 patients that received LD-RT for pneumonia,^19^ Scott noted that patients frequently experienced an initial relief of symptoms, followed by a reduction in fever 12 to 24 hours post-treatment. Mortality rates of 34 patients not receiving LD-RT were used to provide control. A mortality rate of 19.5% was recorded in the treatment group, while the control group had a mortality rate of 44.1%. The patients were monitored for adverse radiation effects; no toxicity events were recorded in the treatment group. The aforementioned control and treatment group mortality rates are illustrated in Figure 2.

It is noteworthy that in all studies, the reported toxic effects of LD-RT were minimal. This is an important clinical benefit when considering—and as compared to—other contemporary (systemic) therapeutics. Additionally, these studies reveal a distinct pattern of clinical success. LD-RT reduced mortality rates, rapidly alleviated symptoms, and substantially shortened and reduced the severity of pneumonia. We opine that these results, although certainly suggestive of LD-RT efficacy, should not be taken at face value. In the 1940s, explanation of putative mechanisms for these results were limited, full extent of risk was not ascertained, and the paucity of such data undergird the need for additional, more detailed (and well-controlled) studies. This lack of evidence was also noted by the researchers themselves, who emphasized the necessity of further research, and advocated use of other, more established treatments before resorting to LD-RT. Their caution against unwarranted causal inference serves as a valuable milestone in calls for systematic, evidence-based reasoning.

Despite the early successes and apparent promise of LD-RT, its continued use to treat pneumonia diminished following the introduction of penicillin, and public concerns about the effects of radiation subsequent to the dropping of the atomic bombs and the progression of the Cold War.^20^ Therefore, in all likelihood, the use of LD-RT for pneumonia has fallen out of favor and had been largely forgotten. But the current COVID-19 crisis has fostered needs for more effective treatment of the most severely afflicted patients, and has generated renewed interest in the putative benefit and value of LD-RT.

## COVID-19 and Immunomodulation Through LD-RT

**Figure 3.**
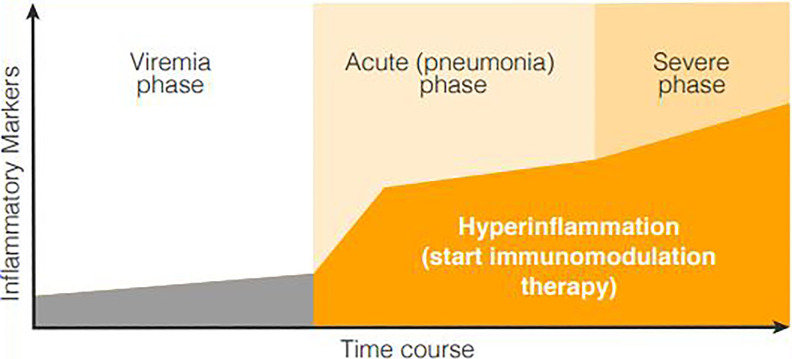
Schematic chart of the COVID-19 course, phases and progressive inflammation.^21^

The clinical course of COVID-19 entails 3 phases^21^: the viremia phase; acute phase (viral pneumonia); and either a recovery phase or severe/critical phase (see Figure 3). Given an appropriate immune response during the first 2 phases, there is a high likelihood that the patient will clear the virus and recover. However, if the immune response is excessive, the severe phase and criticality can occur, which is characterized by a hyperinflammatory state associated with increased mortality.^22-24^ In this severe phase, a systemic inflammatory response develops that (characteristically is) the result of cytokine storm wherein there is over-production and systemic release of proinflammatory cytokines.

LD-RT is known to affect both immune and endothelial cells. *In vitro*, LD-RT induces decreased adhesion of leukocytes to endothelial cells, and apoptosis (when administered at doses of 0.1-0.5 Gy).^26^ This decreased adhesion may be caused by a reduced expression of E-selectin, an endothelial cell adhesion molecule (the production of which has been shown to be decreased *in vitro* after exposure to 0.3-0.5 Gy).^27^ In a mouse model of collagen-induced arthritis, an increase in regulatory T cells, which are capable of dampening immune responses, were observed after treatment with LD-RT.^28^

LD-RT has been shown to mitigate the proinflammatory effects of macrophages in murine studies. Prior to stimulation with lipopolysaccharide and interferon (IFN)-γ, LD-RT reduced the secretion of nitric oxide by macrophages *in vitro*.^29^ Furthermore, proinflammatory cytokine production by macrophages in response to stimulation with lipopolysaccharide *in vitro* was shown to be suppressed by LD-RT.^30^ Similarly, the secretion of reactive oxygen species by macrophages was depressed by LD-RT when administered at doses between 0.3 and 0.6 Gy *in vitro*.^31^

Calabrese et al. have suggested that LD-RT induces polarization of M1-type macrophages to the anti-inflammatory M2-type.^32^ This polarization distribution is likely not absolute, but rather represents a combinatory state of differing macrophage phenotypes. The M1- to M2- phenotypic conversion may be important to clinical outcomes of inflammatory disease, as the M2-type macrophages secrete the anti-inflammatory cytokines, interleukin (IL)-10 and TGF-β1 and suppress the proinflammatory cytokine IL-6.^33,34^

Patients with critical COVID-19 illness (i.e.- hyperinflammation) have been shown to have high levels of cytokines, particularly IL-6^22^ as constituent to cytokine storm. Downregulating proinflammatory cytokines in COVID-19 hyperinflammation could prevent the onset of a critical stage of disease. LD-RT has been shown to reduce IL-6 *in vitro, but also demonstrated downregulation of additional proinflammatory cytokines that are involved in the pathological process of hyperinflammatory COVID-19* ^25,35-37^, *namely,* tumor necrosis factor (TNF)-α^12,34^, IL-1β^12,34^, IL-8^38^ and INF-γ^39^ (Table 1). Therefore, we posit that LD-RT may be of benefit in reducing cytokine storm-induced hyperinflammation and potentially could mitigate or prevent the severe/critical phase of COVID-19, inclusive of ARDS.

**Table 1.** ProInflammatory Cytokines.

Increased in COVID-19	Reduced with LD-RT
TNF-α [25*,34*]	TNF-α [12*,36**]
IL-1β [35*]	IL-1β [12*,36**]
IL-6 [34*,35*]	IL-6 [36**]
IL-8 [35*]	IL-8 [37**]
INF-γ [34*,38*]	INF-γ [39*]

* in vivo. **in vitro.

## Consideration of Risk When Using LD-RT for Severe COVID-19

Important to our view of the potential value of LD-RT is consideration of the potential burden and risk(s) of this intervention. First, according to current low dose linear biostatistical modeling, the direct induced (carcinogenic) risk of LD-RT at doses of 0.5 to 1.0 Gy, is well below the reported risk of spontaneously occurring carcinogenesis.^40^ Thus, the use of LD-RT appears to pose minimal risk in this regard. When considering the risk of secondary tumor, linear modeling-based analysis reveals that a single fraction of 0.5 Gy administered to the thorax induces risk of approximately 1% after 15-20 years.^41^ Additionally, a recent study investigated the risk and occurrence of breast cancer in 158 women who received cumulative average dosing of 7 Gy LD-RT for the treatment of shoulder-related diseases. Follow up after 21 years revealed no indication of secondary breast cancer due to LD-RT.^42^ As well, acute tissue injuries and/or bone marrow suppression, both known consequences of radiation therapy, were determined to be very unlikely at this dose.^43^

Patients with COVID-19 and ARDS that are admitted to the ICU receive intensive supportive therapy through mechanical ventilation (although this may not be not suitable for some patients). For hospitalized patients with severe COVID-19, the mortality rate is 13-25% at day 28 of the illness.^44,45^ This substantial death rate at day 28 is higher than any known cancer, yet, cancer patients are frequently and routinely treated with radiotherapy doses that are much higher than LD-RT dosage recommended here.

## Recent Clinical Trials Using LD-RT for Severe COVID-19

Two recent pilot-studies have investigated the risks involved in LD-RT, and published preliminary findings about the effectiveness of this treatment on small numbers of COVID-19 patients. The RESCUE-1-19 trial was first to demonstrate that a single 1.5 Gy LD-RT, of 10 minutes or less in duration, was safe (in the first 5 patients treated^46^). No acute dermatologic, pulmonary, cardiac, GU or GI toxicities were observed. Of note was that, 4 (of the 5) patients showed significant clinical improvements and did not require supplemental oxygen after a mean of 1.5 days following LD-RT. This report, initially released in MedRxiv, has subsequently been peer-reviewed and published.^47^

**Figure 4.**
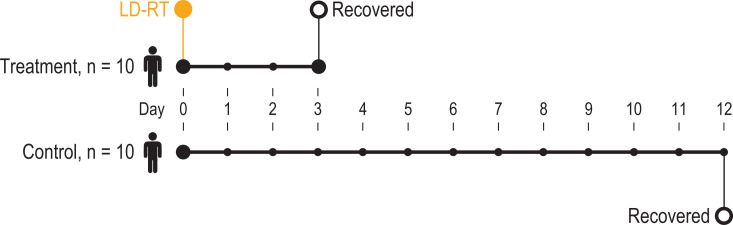
Median time to clinical recovery of patients treated with LD-RT versus controls. A subject was assigned a recovered status when one of the three ordinal categories was satisfied; (1) Not hospitalized, no limitations on activities; (2) Not hospitalized, limitation on activities and/or requiring home oxygen; or (3) Hospitalized, not requiring supplemental oxygen.

The results of this 5-patient pilot-study prompted extension of the trial to confirm efficacy in an additional 5 patients (i.e.- total n = 10 patients). Eligible patients were hospitalized, showed radiographic consolidations and received supplemental oxygen. All 10 COVID-19 patients were treated with whole-lung LD-RT, and their outcomes were compared to age- and comorbidity-matched controls with at least a 28-day follow-up.^48^ The median age of the treatment and control cohorts was 78 years for LD-RT vs 75 for control. The LD-RT cohort appeared to have a poorer clinical prospect, as evidenced by a lower median Glasgow Coma Scale, higher comorbidity index, and a lower median baseline P: F ratio. The control cohort received best currently accepted supportive care, and 6 of these patients were given an alternative COVID-19 experimental therapy. Despite this, the median time of recovery for the control cohort was 12 days versus a 3-day median recovery of the LD-RT group (p = 0.048) (see Figure 4). Patients receiving LD-RT demonstrated improvements in radiographically identified lung consolidations by day 7-21 (interpreted by a radiologist who was blinded to the nature of treatment(s) patients had received). Notably, 9 of 10 patients (90%) in the LD-RT group showed radiographically identifiable improvements vs 4/9 in the control group (44%), p = 0.03. Of particular interest was the observed reduction of the inflammatory markers C-reactive protein (CRP) and IL-6 in the treatment group, which further strengthens support for LD-RT acting by suppressing the inflammatory response. Within 24 hours after LD-RT, 1 patient experienced an acute upper gastrointestinal toxicity reaction (i.e.- nausea). Another LD-RT-treated patient presented with increasing oxygen dependence, requiring high-flow oxygen support for 4 days following treatment, progressed to coagulation, cardiac, and renal abnormalities, and required intubation 5 days following LD-RT, and ultimately died on day 15. The authors concluded that the safety and efficacy of LD-RT warrant further investigation, and toward these ends have commenced a larger phase 3 trial.^49^

A second pilot study included 5 severe phase COVID-19 patients (median age 69), without matched controls.^50^ These patients were hospitalized, demonstrated radiographic consolidations, and required supplemental oxygen. All 5 patients were treated with a single-dose LD-RT, 0.5 Gy, and were followed for 5-7 days to evaluate treatment response, clinical outcomes, and potential toxicity. One of these patients died. Of the 4 patients treated, clinical improvements were reported on the first day after treatment, with demonstrated reduction in CRP and IL-6 following the course of clinical improvement in LD-RT treated patients, with no acute toxicities noted. The authors also concluded that while apparently effective, additional trials are needed to further investigate the role of LD-RT for hospitalized COVID-19 patients.

## Discussion

Historically, LD-RT has been successfully used to treat pneumonia cases, in some instances providing a lifesaving immunomodulating therapeutic option. Although the use of LD-RT has largely been forgotten, such historical success—coupled to the positive outcomes of recent clinical studies—prompt our call for consideration of LD-RT for those severe/critically ill COVID-19 patients in a hyperinflammatory state. *In vitro* and *in vivo* research demonstrating anti-inflammatory mechanisms of LD-RT further support its consideration for use in suppressing the extent and effects of cytokine storm in COVID-19 patients. The 2 recent small-n trials reported a total of 15 severe COVID-19 patients treated with LD-RT. The preliminary success and the clinical benefits observed in these trials may offset the minimal potential for long-term, secondary (cancer) risks in these patients. LD-RT appears to effectively reduce the hyperinflammatory state, and therefore warrants additional, larger scale randomized controlled trials to further assess the viability and value of this intervention. As of this writing, multiple research centers and hospitals have initiated such trials and have begun evaluating the efficacy of LD-RT for COVID-19 in greater detail.

To be sure, the disease process caused by SARS-Cov-2 is complex, and the effects of LD-RT on the hyperinflammatory states of COVID-19 patients may be too subtle to prevent fatal outcomes in all severe/critical cases. However, a pleiotropic immunomodulating effect of LD-RT could be beneficial, especially in the most vulnerable COVID-19 patients. Cognizant of concerns about the possible carcinogenic effects of radiation exposure, we suggest that the long-term mortality risk of LD-RT is expected to be very low, as based upon the studies cited herein.

At this time, therapeutic options for COVID-19 are limited. Currently advocated precepts of medical ethics^51-57^—and policies^58,59^—support that non-indicated, but evidence-supported and -fortified interventions may be used if no other treatment options exist for the alleviation or cure of a disease. Therefore, we conclude, as based upon the evidence provided above, that LD-RT may be regarded and considered to be a viable and potentially valuable intervention to decrease cytokine storm-induced inflammatory effects in critically ill COVID-19 patients. We argue that when informed by a demonstrated presence of inflammatory markers and patient characteristics indicative of a worsening moribund (if not likely mortal) prognosis, the clinical decision to use a single-dose of 0.5 -1.5 Gy LD-RT can—in the absence of other available therapeutic options—be regarded as an exercise of humanitarian and exceptional care.

Clinicians considering this treatment should carefully monitor inflammatory markers pre- and post-treatment in order to provide further insight that will be essential to validating the effectiveness—and putative mechanism(s)—of LD-RT. At present, those COVID-19 patients progressing to a severe state, other options are at least few, and at worst, evidently ineffective and unsuccessful. We believe that the needs of these patients provide ethical ground for exploring the use of other, more capable interventions. Indeed, as the adage informs, necessity is the fountainhead of invention and innovation. Our hope is that history may provide us with evidence to foster our current and future clinical ingenuity in treating this novel threat to patients’ and public health to be used in further research.
